# Ophthalmology in Persian medicine

**Published:** 2014-12-03

**Authors:** Seyed Mahmoud Tabatabaei, Nastaran Sabetkish, Seyed Mohammad Ali Tabatabaei

**Affiliations:** 1 Professor, Medical Ethics and History of Medicine Research Center, Tehran University of Medical Sciences, Tehran, Iran;; 2 Pediatric Urology Research Center, Section of Tissue Engineering and Stem Cells Therapy, Children’s Hospital Medical Center, Tehran University of Medical Sciences, Tehran, Iran;; 3 Postgraduate Student, Department of Periodontics, Dental Faculty, Babol University of Medical Sciences, Babol, Iran.

**Keywords:** Rhazes, Avicenna, ophthalmology, Persian medicine, al-Hawi

## Abstract

Despite the fact that ophthalmology is one of the foremost branches of medicine, conceptualization of the structure and function of the eye barely advanced in ancient Western civilizations. At the early recovery of Persian civilization (9^th^ century AD) after the extinction of the Sassanid Empire (7^th^ century AD), translations of Greek medical textbooks played an important role in the development of medicine and the emergence of great Persian physicians such as Rhazes, Avicenna and others. Rhazes was a leading Persian physician whose medical teachings have as yet not been thoroughly explored. In addition to numerous books and articles in various fields, he authored a great medical Encyclopedia (al-Hawi al-Kabir) in 25 volumes. In this article, we are going to compare Rhazes’ particular viewpoints about ophthalmology with those of other famous Persian physicians and some recent essays and textbooks.

For this purpose we reviewed Rhazes’ second volume of al-Hawi that is dedicated exclusively to ophthalmology and contains some major topics of ophthalmology including anatomy, physiology, pathology, diseases, disorders and treatments. Important themes were carefully extracted and compared with the tenets of modern ophthalmology.

After collating Rhazes’ viewpoints with the latest findings in this field, it was concluded that he had brilliantly written about the signs and symptoms, etiology and treatment of many eye disorders more than a thousand years ago. The amazing point is that there was no accurate equipment at the time to help him in his investigations.

This study proved that Rhazes’ theories conform to recent knowledge about ophthalmology in many aspects, and could therefore be the subject of further investigations.

## Introduction

Considering the importance of vision as a basic human faculty, it seems that the hygiene of the eye, ocular diseases and their treatments were among the oldest concerns in the history of medicine. Hippocratic medicine greatly influenced the advancement of all branches of medicine, including ophthalmology. The influence commenced in Rome where Galen helped develop this area of medicine (1, 2). According to historical documents, Persia had a thousand year history in medicine (3-5). In the 9^th^ century AD, when Persian civilization began to recover after the fall of the Sassanid Empire two centuries ago, medical literature from different civilizations including Greece, India and China reached the Arab world and Persia. The most important books from the point of view of scientific content and clinical application were Dioscorides’ and Galen's medical references. These works were translated mainly by leading Christian physicians such as Stephen, Honein (Johan), and others who lived in the Islamic capital Baghdad in the mid 9^th^ century AD. These translations helped the evolution and development of medicine In Arab countries and Persia, and resulted in the emergence of great Persian physicians such as Rhazes, Avicenna and others (6). 

Muhammad ibn Zakariya al-Razi, known in Western literature as Rhazes, was one of the greatest and most influential Persian physicians. Using his great knowledge of Persian, Greek and Indian medicine as well as his personal observations and discoveries, he wrote about 180 books and articles in various fields of science (7). Al-Hawi, which is in fact a medical encyclopedia written in 25 volumes in the 10^th^ century AD, is one of these books. It is a compilation of knowledge gathered from other works as well as Rhazes’ own theories and clinical experiences. In the second volume of al-Hawi, which is approximately 400 pages, ophthalmology is discussed exclusively (8). Translation of this encyclopedia into Persian first began in 1989. Overall, four volumes were published, as well as a summary in three volumes entitled Synopsis of al-Hawi (9, 10). 

In this article, we aim to investigate the theories and clinical experiences of Rhazes, Avicenna and some other Persian physicians by juxtaposing their viewpoints against contemporary scientific studies in the field of ophthalmology.

***Rhazes' Viewpoints about Diseases and Disorders of the Eye***

**Disorders of the Eyelids**

*Adhesion of eyelids* **:** According to Rhazes, this condition is categorized in two separate groups:

     i. Adhesion of eyelids to any part of the eyeball that may occur after removal of pterygium or healing of sores. It seems that this phenomenon is known as symblepharon in modern ophthalmology (9-11).

     ii. Adhesion of a part of one eyelid to another. Rhazes explains that this condition could happen during sleep or as a result of conjunctivitis recovery. It seems that this phenomenon is known as symblepharon in modern ophthalmology as well (9-11). 

*Ectopic cilia* **:** Rhazes describes a condition in which cilia are grown along the edge of the eyelid toward the inside causing irritation, abrasion and pain (9, 10). In recent studies, it has been clarified that distichiasis is the abnormal growth of lashes from the orifices of the meibomian glands, while trichiasis is a normally grown cilium that is misdirected toward the eye (12).

     *Pediculosis*: It is remarkable that Rhazes mentions the major cause of this disease to be a low level of personal hygiene (9, 10). According to modern literature, there is a significant association between pediculus humanus corporis or body lice and poor hygiene (13-16).

**Disorders of the Conjunctiva**

*Conjunctivitis* **:** Rhazes defines this disease as the acute inflammation of the conjunctiva accompanied by redness or purulent discharge. He categorizes the risk factors in three groups (9, 10):

     i. Exposure to sunlight, heat, dust or greasy substances.

     ii. A hot bath in patients who are prone to developing conjunctivitis. Rhazes emphasizes that vasodilation due to a hot bath can cause this type of conjunctivitis.

     iii. Red roses.

The first and third categories are well-known examples of allergic conjunctivitis where a history of itching may help with the diagnosis (17 - 20). In the first category, dry eye is induced by an underlying condition that triggers allergic conjunctivitis, while in the third category; allergic conjunctivitis is induced by an allergen.

According to Rhazes, there are four types of conjunctivitis (9, 10):

     1) In the first type a well-known risk factor such as sunlight, heat, dust, smoke or a greasy substance causes the disease. This condition is known today as irritative conjunctivitis. Rhazes considers this type of conjunctivitis to be so slight that it can easily be cured through avoiding the agents mentioned above, and this is exactly how this type of conjunctivitis is treated nowadays (20).

     2) Rhazes believes that an acute inflammation of the conjunctiva could cause the second type of conjunctivitis (9, 10). This is known as epidemic keratoconjunctivitis (EKC) and is associated with etiology of adenovirus. In this type of conjunctivitis a remarkable inflammation affects the conjunctiva and makes it irritated.

     3) Rhazes declares that in both the above-mentioned types of conjunctivitis, harmful discharge might cause inflammation, pain, thickness, itching, and redness of the conjunctiva. The third type may be accompanied by lacrimation, excessive redness, and laborious lid movements. Modern studies have established the association of ocular itching with allergic conjunctivitis after exposure to certain allergens (17-20). Stringy or ropy discharge due to conjunctivitis has also been mentioned in a number of articles (20). 

     4) The last type of conjunctivitis mentioned by Rhazes is cyclic and may occur every day or in intervals (9, 10). This subcategory is known today as seasonal conjunctivitis or vernal keratoconjunctivitis in which patients may experience remissions and exacerbations (20). 

*Pterygium* **:** Rhazes describes this disorder as a condition in which there is an excessive growth in conjunctiva, usually starting from the inner corner of the eye and extending on the surface of cornea and eventually covering the pupil. It can be soft or hard and its color may be yellow or red (9, 10). In recent studies, it has been indicated that pterygium is a kind of conjunctive epithelial hyperplasia with a high rate of proliferation originating from the bulbar conjunctiva over the cornea (21-25). Moreover, It is now classified as fibrous (yellow and hard) or vascular (red and soft). Accordingly, Rhazes’ classification is similar to the modern categorization. Pterygium without inflammation has a white or yellow appearance, but in the presence of inflammation, the condition is called pterygitis and is red in color. 

*Keratoconjunctivitis:* Rhazes also defines a condition in which a thin smoky layer of protuberant blood vessels overlie the cornea and conjunctiva and make the eyeball red and hard. In most cases symptoms such as lacrimation, watery discharge, itching and inflammation arise and the patient cannot see objects in sunlight or other lights (9, 10). This may be a rare condition known today as atopic keratoconjunctivitis in which patients suffer from corneal vascularization, intense itching and redness in the eyes, mucosal discharge, and difficulty in opening their eyes outdoors in prolonged cases (20, 26). Considering the fact that vernal keratoconjunctivitis is more prevalent in Iran climate, Rhazes may have described a condition that induces severe papillary conjunctivitis, giant papillae, gelatinous thickened accumulation of tissue around limbus, and presence of discrete whitish raised dots along the limbus (Tranta's spots) (27).

**Disorders of the Cornea**

*Corneal abrasion* **:** Rhazes states that the cause of these abrasions might be the collision of iron, wood or other foreign objects with the cornea, or the inappropriate use of ophthalmic medication (9, 10). 

*Corneal melting* **:** Rhazes believes that either the first layer or all three layers of the cornea might be affected in this disorder. It is noteworthy that he divided the cornea into three layers (9, 10) without access to the complex equipment that exist today.

Rhazes describes a disorder in which white or black lesions appear when discharge settles between the layers of cornea (9, 10). According to recent data, this condition is known as superficial punctate keratitis and is characterized by a breakdown of the corneal epithelium in a pinpoint pattern. Red eye, foreign body sensation, tearing, photophobia, and a burning sensation are among the non-specific symptoms (9, 10).

**Disorders of the Iris**

Rhazes explains that the pupil is opened and closed with the help of a muscle, and visual disorders occur when this muscle is damaged. He classifies iris disorders in four groups (9, 10):

*Widening and narrowing of the pupil* **:** A widened pupil may be the result of an expansion of the iris, or a loose iris that cannot contract properly. The narrowing of the pupil on the other hand may be due to iritis, opium use, or excess light. 

*Protrusion of the iris* **:** According to Rhazes, the iris might protrude through the damaged part after corneal ruptures.

*Iris rupture* **:** Rhazes declares that the first complication of iris rupture is that an aqueous substance spills out and causes other complications. According to modern ophthalmology, iris rupture may induce glaucoma due to a condition named angle recess.

*Nyctalopia and hemeralopia* **:** Rhazes points out that some patients do not have accurate vision during the night, and some experience this problem in the daytime (9, 10). Today, these patients are known as nyctolopic and hemeralopic respectively. However, these conditions are known to occur as a result of retinal photoreceptors disorder. 

**Impairment of Eye Muscles**

Rhazes explains that when a muscle located near the root of a nerve weakens, the eye will be drawn outward (9, 10).

According to Rhazes, if the weakness is insignificant, the protuberance of the eye will be negligible and vision will not be affected. These findings refer to Phoria or latent deviation (as opposed to manifest deviation). However, if the weakness is significant, it may even lead to blindness. Rhazes believes this condition to be the result of a shortening or weakening of the muscles situated on one side of the eye that will terminate in dislocation of the eyeball and its traction to the opposite side (9, 10). These findings refer to amblyopia induced by strabismus, or eye deviation. According to Rhazes, if the deviation of the eyeball is upward or downward, diplopia occurs (9, 10).

**Disorders of the Optic Nerve**

In cases of blindness or sight diminution where no obvious damage is found during eye examinations, Rhazes recommends that optic nerve or even brain damage be taken into consideration. His categorization of the factors causing optic nerve disorders is remarkable (9, 10):

     i. Constitutional changes

     ii. Nerve compression

     iii. Inflammation

     iv. Tumors

     v. Decomposition of organs

     vi. Optic nerve ruptures

*The Importance of the Optic Nerve*

     Rhazes considers optic nerve damage or rupture to be the most important risk factor for blindness or visual disorders (9, 10). According to his theory, when blindness is accompanied by protrusion of the eye, optic nerve has been ruptured or protracted excessively. Today, it is known that thyroid ophthalmopathy may be accompanied by optic neuropathy. Rhazes specifies that eyeball protrusion and limited vision are symptoms of flabby eye muscles.

**Eye Cancer**

Rhazes states that when a malignancy occurs in one of the eye layers, patients experience protraction, redness, and severe pain especially during eye movements. According to Rhazes, this condition is incurable, but pain relief should be offered. He adds that the pain is aggravated by hiking and considerable body movements that are usually followed by extensive headaches. He also states that vein dilation can develop in eye cancer. 

Other symptoms also associated with eye cancer in modern ophthalmology include diluted and irritative eye discharge and lack of appetite (9, 10).

**The Origin of Eye Disorders**

Rhazes categorizes the risk factors in two major groups (9, 10):

     1) The first category consists of extracranial causes, most importantly trauma. This category is divided into three subgroups:

     a) Traumas that cause a dramatic disturbance in the function of eye muscles. In these types of injuries, patients do not suffer from any kind of ocular dysfunction or vision loss, but the ability to move the injured eye is decreased. Blowout fracture is one of the traumas that can cause this kind of disturbance. In recent articles it has been mentioned that inferior and medial rectus muscles can be pathologically involved in orbital floor fractures, which may be followed by permanent damage of the neuromuscular complex. Decreased extraocular motility is a well-known consequence of such fractures (28). 

     b) Traumas in which the optic nerve is torn. These injuries cause total blindness. In modern literature, blunt or penetrating trauma and especially indirect pressure to the head that can cause optic neuropathy are mentioned as a cause of permanent visual loss (29).

     c) In some other cases, trauma causes a hemorrhage in the eye. As mentioned in some articles, acute retrobulbar hemorrhage that can occur after retrobulbar injections and trauma to the orbit is known as a sight threatening condition. They may also cause subconjunctival, vitreous, and retinal hemorrhages. Vitreous and retinal hemorrhages decrease vision while retrobulbar hemorrhages induce compressive optic neuropathies (30-32).

     2) In the second category Rhazes states that intracranial growths, encephalitis or inflammation of the optic nerve can terminate in complications mentioned below: 

     a) Damage of the optic nerve in the brain (cortical blindness).

     b) Damage to the nerves sending signals to the eyeballs (muscular or nerve palsies, like cranial nerves IV, III, or VI).

Some recent articles assert that acute demyelination of the optic nerve due to multiple sclerosis or an isolated neurologic disease can result in visual impairment (33-37).

**Viewpoints of Avicenna and other Persian Physicians about Ophthalmology**

In the chapter dedicated to ophthalmology in his famous medical textbook Canon, Avicenna describes the anatomy, physiology, pathology, diseases and treatments of the eye (38). Considering that Avicenna lived about 80 years after Rhazes, he would have access to Rhazes’ works and therefore there is no major difference between their views about ophthalmology.

Other physicians such as Ali ibn Sahl al-Tabari (9^th^ century AD), Ali ibn Abbas Alahwazi, also known as Haly Abbas (10^th^ century AD), and Syed Ismail Jurjani (13^th^ century AD) are Persian physicians who have described various topics of ophthalmology in their medical textbooks (1, 39, 40). Their viewpoints and descriptions are not as comprehensive and methodical as Rhazes. The viewpoints of Avicenna and the above-mentioned Persian physicians about ophthalmology can be a topic of research for future articles.

## Conclusion

Reviews of ancient Persian medical textbooks indicate that the most noteworthy and all-inclusive textbook of ophthalmology has been written by Rhazes. The definitions, divisions, and classifications of ophthalmic diseases and disorders have been offered with remarkable precision in this work and have not lost their scientific value even after eleven centuries. Issues that have been discussed in this article are excerpts from Rhazes’ medical encyclopedia that was written in over 400 pages. 

It may be added that by reviewing the works of traditional physicians and using advanced technology, modern medicine has been able to identify the topics of ophthalmology defined by Persian physicians. It should be noted that these contents have never lost their historical value, and contain important educational and clinical points that are similar to concepts in modern ophthalmology.
